# Comparing the Anterior-Based Muscle-Sparing Approach with the Direct Anterior Approach in Hip Arthroplasty: A Systematic Review and Pairwise Meta-Analysis

**DOI:** 10.3390/medicina59081390

**Published:** 2023-07-29

**Authors:** Jae Suk Chang, Min Wook Kang, Dong Hwan Lee, Ji Wan Kim, Chul-Ho Kim

**Affiliations:** 1 Department of Orthopedic Surgery, National Police Hospital, Seoul 05715, Republic of Korea; jschang3525@gmail.com (J.S.C.); npng4eve@gmail.com (M.W.K.); gopress@hanmail.net (D.H.L.); 2 Department of Orthopedic Surgery, Asan Medical Center, University of Ulsan College of Medicine, Seoul 05505, Republic of Korea; bakpaker@hanmail.net

**Keywords:** anterior-based muscle-sparing approach, direct anterior approach, anterolateral approach, hip arthroplasty, meta-analysis

## Abstract

*Background and Objectives*: The anterior-based muscle-sparing (ABMS) approach, which utilizes the interval between the tensor fasciae latae posteriorly, offers several advantages, such as the reduced risk of nerve injury and the freedom to choose various implants. Herein, we aimed to compare the outcome of ABMS to the direct anterior (DA) approach using pairwise meta-analysis techniques. *Materials and Methods*: A systematic search of the MEDLINE (PUBMED), Embase, and Cochrane Library databases was performed for studies published up to 7 June 2023, which compared the ABMS approach with the DA approach for hip arthroplasty. We compared (1) perioperative outcomes (operation time, visual analog scale (VAS) score, total opioid consumption, length of hospital stay (LOS), and the number of patients discharged to their homes); (2) postoperative complications (neuropraxia/nerve injury, dislocation, surgical site infection, intraoperative fracture, and reoperation rate); and (3) implant position (cup inclination, cup anteversion, and stem alignment). *Results*: Ten studies were eligible for meta-analysis, including 1737 patients who underwent hip arthroplasty with the ABMS approach and 1979 with the DA approach. The pooled analysis showed no differences in all outcome variables, including perioperative outcomes, postoperative complications, and the implant position between the two surgical approaches. *Conclusions:* In current meta-analysis, the ABMS approach demonstrated comparable results to the conventional DA approach in terms of both clinical and radiologic outcomes as well as postoperative complications. Furthermore, the ABMS approach has the advantage of a broader indication and fewer limitations in terms of the surgical position compared to the DA approach. Therefore, the ABMS approach can be even more beneficial as an option within MSA, surpassing the utility of the DA approach.

## 1. Introduction

Hip arthroplasty (HA) is one of the oldest and most renowned surgeries in the field of orthopedics, often referred to as the “operation of the century,” with a history spanning over 100 years [1,2]. This surgery has demonstrated its effectiveness in alleviating pain and restoring mobility.

In recent decades, a multitude of surgical approaches have been introduced and refined with the aim of improving surgical outcomes following HA. The emphasis has been directed towards the mitigation of postoperative pain, reduction in postoperative rehabilitation durations, and attainment of heightened functional recuperation. Consequently, in recent years, there has been a growing interest in less invasive muscle-sparing approaches (MSAs), particularly in the field of adult hip reconstruction [3].

The MSA to access the hip joint is not a recent development. It has roots in some of the oldest approaches to the hip joint, namely the Smith-Petersen and Watson-Jones approaches. The direct anterior (DA) approach for HA, one of the most commonly used MSAs today, was first introduced by Judet in 1947 [4,5]. However, the MSA did not gain widespread acceptance for a while due to technical difficulties and the limited availability of specialized surgical instruments. Instead, the posterior approach, considered easier to learn, became the dominant technique worldwide. Nonetheless, the posterior approach has inherent problems such as a dislocation risk and sciatic nerve injury [6], necessitating a more favorable approach. Consequently, there has been a surge in interest in anterior-based approaches, leading to an increased adoption of the MSA.

Among the various MSAs, the DA approach, based on the Smith-Petersen approach and utilizing Heuter’s interval for access [7], has gained significant popularity in recent years. However, there are some drawbacks to consider. First, the relatively medial location of the skin incision warrants special caution regarding the risk of lateral femoral cutaneous nerve injury. Additionally, the technique has a long learning curve, and there are limitations in terms of implant selection [8,9]. Furthermore, patients with a higher body mass index are frequently excluded from selection for the DA approach due to difficulty with exposure, implant placement, and surgical wound management [10]. 

The anterior-based muscle-sparing (ABMS) approach, also called the anterolateral (AL) approach, abductor sparing/muscle sparing/mini AL approach, or ABLE approach, represents an alternative MSA that addresses some of the drawbacks associated with the DA approach [3]. It utilizes the interval between the tensor fasciae latae posteriorly, building upon modifications of the previously described Watson-Jones approach. Compared to the DA approach, the ABMS approach offers the advantage of a relatively more lateral incision, potentially reducing the risk of nerve injuries. It can be performed in both supine and lateral positions, facilitating a shorter learning curve and providing greater freedom in implant choice [11]. 

Despite its potential advantages, there is a paucity of research on the specific benefits and drawbacks of the ABMS approach, as well as a lack of comparative analysis regarding the outcomes of the ABMS approach compared to the DA approach [3]. Therefore, this study aims to analyze the outcomes of the ABMS approach versus the DA approach using the pairwise meta-analysis technique.

Our hypothesis is that the ABMS approach will be comparable to the DA approach in terms of clinical outcomes, postoperative complications, and radiologic outcomes. Additionally, we believe that the ABMS approach offers the advantages of a short learning curve, greater surgical freedom in positioning, and more flexibility in implant selection compared to the DA approach. Therefore, overall, the ABMS approach could be deemed a more advantageous alternative for surgeons who are less familiar with the DA approach.

## 2. Materials and Methods

The present study was conducted in accordance with the Revised Assessment of Multiple Systematic Reviews (AMSTAR) and Preferred Reporting Items for Systematic Reviews and Meta-Analyses (PRISMA) guidelines [12,13]. While this analysis involved human participants, both ethical approval and the acquisition of informed consent from participants were not required because all data were based on previously published studies and were anonymously analyzed without any potential harm to the participants.

### 2.1. Literature Search

Following the referenced guidelines, we systematically searched MEDLINE, Embase, and the Cochrane Library for studies comparing the ABMS approach with the DA approach in hip arthroplasty. Using an a priori search strategy, we identified articles published up to 7 June 2023, without any language or publication year restrictions. The search terms included synonyms and terms related to hip replacement, the ABMS approach, and the DA approach. The full search strategies and results for all databases are presented in Supplementary Table S1. After the initial electronic search, we manually searched for relevant articles and their bibliographies.

### 2.2. Study Selection

Two board-certified orthopedic surgeons (J.S.C. and C-H.K.) who worked as faculty members at an academic medical center independently selected the articles for a full-text review based on the titles and abstracts of the studies. In cases where the abstracts provided insufficient information, the full article was reviewed.

This meta-analysis was designed as a pairwise meta-analysis. Studies were included based on the “Population, Intervention, Comparator, Outcome, Study design” (PICOS) criteria [14]: (1) patients who underwent hip replacement (population), (2) total hip replacement using the ABMS approach (intervention), (3) the DA approach (comparator), and (4) reported outcomes. 

Careful consideration was given to excluding studies with inaccurate definitions of the term “ABMS.” While most papers correctly used the term “anterolateral (AL) approach” as a synonym for ABMS, some inaccurately defined the AL approach as the transgluteal/direct lateral approach. Studies on revision hip arthroplasty, non-original articles, unrelated papers, and duplicates from the same investigation group were also excluded. In cases of overlapping study populations, the publication with the largest sample size was selected for the meta-analysis.

Inter-reviewer agreement was assessed at each stage of article selection using κ-values, with predefined categories for agreement strength (κ = 1 corresponded to “perfect” agreement, 1.0 > κ ≥ 0.8 to “almost perfect” agreement, 0.8 > κ ≥ 0.6 to “substantial” agreement, 0.6 > κ ≥ 0.4 to “moderate” agreement, 0.4 > κ ≥ 0.2 to “fair” agreement, and κ < 0.2 to “slight” agreement). We considered the κ-values ≥ 0.6 as acceptable agreement. Disagreements at each stage were resolved through a discussion between the two investigators to reach a consensus. In cases where a consensus could not be reached, a third investigator was involved in the discussion to facilitate resolution.

### 2.3. Data Extraction

For qualitative data synthesis, standardized forms were used to extract relevant information: (1) study design, (2) country of investigation, (3) mean patient age, (4) sex, (5) type of hip surgery, (6) number of patients in the ABMS and DA approach groups, (7) patient position, (8) type of DA approach, and (9) the outcomes investigated.

For the meta-analyses, data extraction focused on (1) perioperative outcomes (operation time, visual analog scale (VAS) score, total opioid consumption, length of hospital stay (LOS), and the number of patients discharged to their homes); (2) postoperative complications (neuropraxia/nerve injury, dislocation, surgical site infection (SSI), intraoperative fracture, and reoperation rate); and (3) implant position (cup inclination, cup anteversion, and stem alignment). When there were missing data among the variables for the intended meta-analysis, we contacted the authors directly to obtain the original data for statistical analysis.

### 2.4. Risk-of-Bias Assessment

The methodological quality of the included studies was assessed using the Methodological Index for Non-randomized Studies (MINORS) [15], a validated tool for the quality assessment of non-randomized studies. The maximum MINORS checklist score for comparative studies was 24. Two independent reviewers performed the quality assessments. Disagreements were resolved through discussion.

### 2.5. Data Synthesis and Statistical Analyses

Standard mean differences (SMDs) with 95% confidence intervals (CIs) were used for continuous data, while odds ratios (ORs) with 95% CIs were calculated for dichotomous data. Heterogeneity was assessed using the I^2^ statistic, with low, moderate, and high heterogeneity defined at 25%, 50%, and 75%, respectively. Forest plots were utilized to present the outcomes, pooled estimates of effects, and overall summary effects of each study. Statistical significance was set at *p* < 0.05. Data were pooled using a random-effects model that has been recommended previously to avoid overestimating the study results, especially in the medical field [16]. A test for publication bias was not conducted due to the recommended requirement of including at least 10 studies in the meta-analysis [17]. All statistical analyses were performed using Review Manager (RevMan), version 5.3 (The Nordic Cochrane Centre, The Cochrane Collaboration, 2014; Copenhagen, Denmark).

## 3. Results

### 3.1. Article Identification

**Figure 1:**
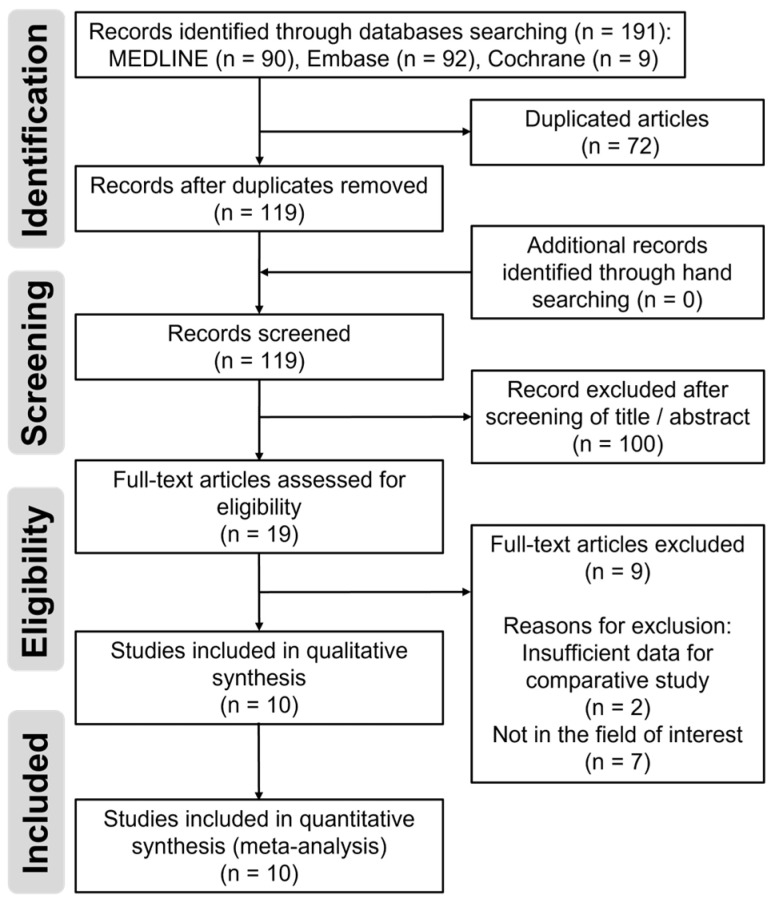
Flow diagram depicting the process of identification and selection of studies included in the meta-analysis.

Details of the study identification and selection processes are summarized in Figure 1. The initial electronic literature search yielded 191 articles. After removing 72 duplicates, 119 articles remained. We could not find any additional articles by manual searching. Of these, we excluded 100 articles after screening their titles/abstracts, and 9 articles were excluded step-by-step after a full-text review. Thus, 10 studies [18,19,20,21,22,23,24,25,26,27] were eligible for qualitative and quantitative data syntheses. The κ-values between the two reviewers were substantial at the title review stage (κ = 0.713), almost perfect at the abstract review stage (κ = 0.800), and in near-perfect agreement at the full-text review stage (κ = 1.000). All κ-values were ≥0.6, which showed the acceptable agreement.

### 3.2. Study Characteristics and Qualitative Synthesis

Of the 10 studies, 7 studies [18,19,20,21,22,26,27] were retrospective cohort studies, 2 studies [23,24] used propensity score matching, and 1 study was a randomized controlled trial. Five studies [18,19,20,21,27] were conducted in the United States, three [23,24,26] in Europe, and two [22,25] in Asia. A total of 3716 hips were included with 1737 hips operated using the ABMS approach and the other 1979 hips operated using the DA approach. The mean age of patients ranged from 61 years to 68.7 years, except for a Swiss study [24], which included patients with a mean age of 85.8 years. All studies predominantly included women who underwent primary HA, with one study [24] exclusively focusing on patients who underwent bipolar hemiarthroplasty. For the ABMS approach, six studies [20,22,24,25,26,27] mentioned the position of the patients. Of these, two studies [20,24] employed the ABMS approach in the lateral decubitus position, while the remaining four studies [18,22,25,26,27] used the ABMS approach in the supine position. For the DA approach, four studies [23,24,25,27] performed the tabled DA approach, and three studies [20,22,26] performed the conventional DA approach. The other three studies [18,19,21] did not specify the type of DA approach used. More detailed information about the outcomes of interest for each study and other relevant details are shown in Table 1.

### 3.3. Risk of Bias Assessment

The MINORS score for assessing the methodological quality had a mean score of 17.5 out of 24 (range: 16–20) (Table 1). When considering the eight primary evaluation parameters, all 10 studies clearly addressed the aim of this analysis (item 1: a clearly stated aim) and appropriately included consecutive patients (item 2: inclusion of consecutive patients). With regards to the study design, all but one study [25] received a deduction for having a retrospective design (item 3: a prospective collection of data). All studies addressed the criteria used to evaluate the main outcomes of interest (item 4: endpoints appropriate to the aim of the study). However, all but one study [25] did not conduct unbiased assessments of their study endpoints (item 5: an unbiased assessment of the study endpoint), with one study [25] partially conducting unbiased assessments. Three studies [23,24,25] were deducted points due to inadequate follow-up durations (item 6: follow-up period appropriate to the aim of the study), while two studies [18,24] received deductions for a loss of follow-up rates (item 7: loss of follow-up rate below 5%). Regarding the prospective calculation of the study size (item 8: prospective calculation of the study size), all studies received point deductions. Points were deducted for three studies [19,20,27] for baseline group equivalence (item 11: baseline equivalence of groups). No deductions were made from the additional criteria domains (items 9,10,12: an adequate control group, contemporary groups, and adequate statistical analyses).

### 3.4. Meta-Analysis

#### 3.4.1. Perioperative Outcomes: Operation Time, VAS Score, Total Opioid Consumption, LOS, and the Number of Patients Discharged to Their Homes

Three studies [19,20,25] compared the operation time between the ABMS approach and the DA approach for hip replacement surgery. In a pooled analysis of 427 patients from the ABMS group and 410 patients from the DA group, there was no difference in operation time between the two groups (SMD, −1.05; 95% CI = −2.31~0.20; *p* = 0.10). The heterogeneity was high (I^2^ = 98%).

Data for the meta-analysis about postoperative day-1 and day-3 VAS scores were extracted from two studies [20,25]. In a pooled analysis of 220 patients from the ABMS group and 218 patients from the DA group, there was no statistical difference in day-1 and day-3 mean VAS scores between the two groups (day-1: SMD, −0.17; 95% CI= −0.35~0.02; *p* = 0.08 and day-3: SMD, −0.20; 95% CI = −0.59~0.19; *p* = 0.31, respectively). The heterogeneity was low in the postoperative day-1 data (I^2^ = 0%) but moderate in the day-3 data (I^2^ = 68%).

Total opioid consumption in the overall postoperative period was compared in two studies [19,20]. In the pooled analysis of 377 patients in the ABMS group and 362 the DA group, there was no difference in total opioid consumption between the two groups (SMD, 0.09; 95% CI= −0.14~0.32; *p* = 0.43). The heterogeneity was moderate (I^2^ = 60%). 

The length of hospital stay was investigated in four studies [19,20,24,25]. There was no statistically significant difference in the LOS observed between the two groups when comparing 585 patients in the ABMS group and 489 patients in the DA group (SMD, 0.02; 95% CI= −0.14~0.32; *p* = 0.43). The heterogeneity was low (I^2^ = 0%).

We could extract the data of several patients discharged to their homes from two studies [20,24]. In a pooled analysis, 161/328 patients were discharged to their homes in the ABMS group, and 151/249 patients were discharged to their homes in the DA group. Statistically, there was no difference between the two groups with low heterogeneity (OR, 1.47; 95% CI = 0.77~2.79; *p* = 0.24; I^2^ = 0%).

**Figure 2:**
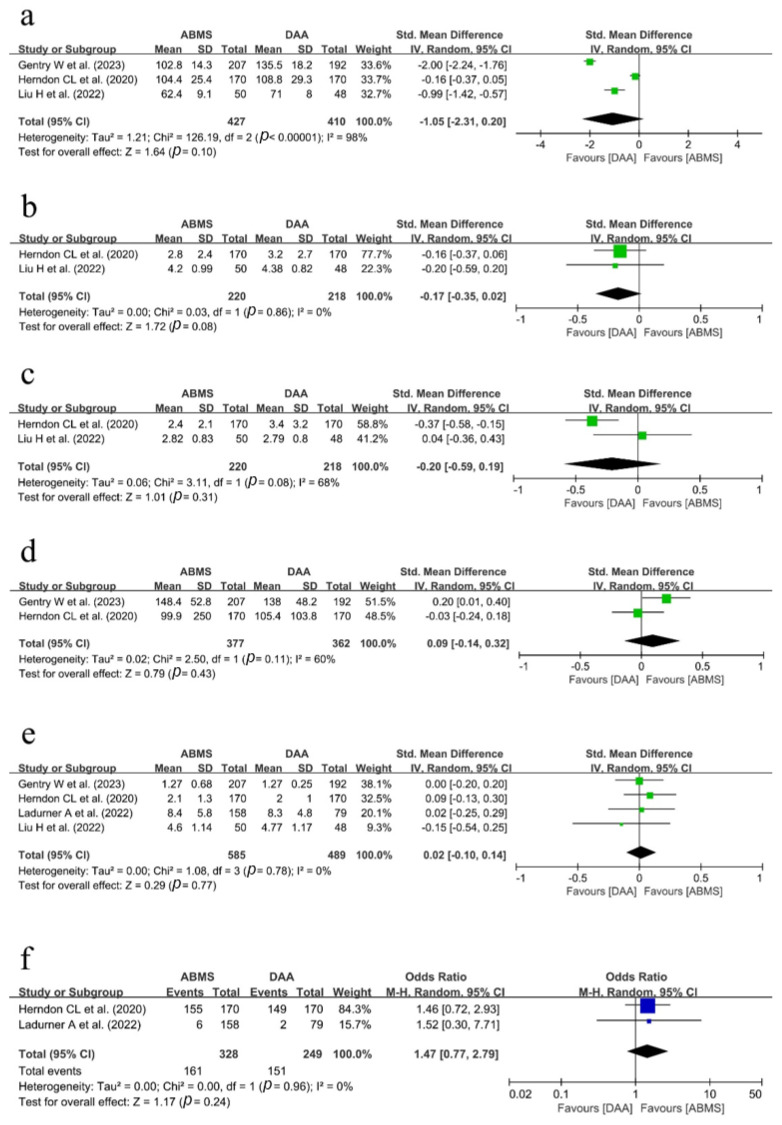
Forest plot showing the perioperative outcomes between the ABMS approach and the DA approach following hip arthroplasty [19,20,24,25]: (**a**) the operation time (minutes), (**b**) VAS score on postoperative day-1, (**c**) VAS score on postoperative day-3, (**d**) total opioid consumption, (**e**) length of hospital stay (days), and (**f**) number of patients who were discharged to their homes.

A forest plot and further details about perioperative surgical outcomes are shown in Figure 2.

#### 3.4.2. Postoperative Complications: Neuropraxia/Nerve Injury, Dislocation, SSI, Intraoperative Fracture, and Reoperation Rate

Four studies [18,22,25,27] compared the incidence of neuropraxia or nerve injury between the two surgical approaches. In a pooled analysis, 11 cases of nerve-related complications were noted among 1918 patients in the ABMS group, and 43 cases were noted among 6028 patients in the DA group. Statistically, there was no difference between the two groups (OR, 0.19; 95% CI = 0.02~1.72; *p* = 0.14), but the heterogeneity was high (I^2^ = 79%).

The postoperative dislocation rate was reported in a total of seven studies [20,21,22,23,24,25,27]. In the ABMS group, there were 8 cases (out of 1446 cases) of dislocation, whereas 16 cases (out of 1689 cases) were reported in the DA group. There was no difference in the dislocation rate between the two surgical approaches (OR, 0.62; 95% CI = 0.20~1.95; *p* = 0.41). The heterogeneity was low to moderate (I^2^ = 32%).

Data on SSIs were reported in a total of seven studies. In the ABMS group, there were 17 cases (out of 1446 cases) of SSIs, whereas 16 cases (out of 1689) of SSIs were reported in the DA group. There was no difference in the SSI rate between the two surgical approaches. Heterogeneity was low (OR, 1.46; 95% CI =0.70~3.05; *p* = 0.32; I^2^ = 0%).

The incidence of intraoperative femoral fractures, including trochanteric fractures and calcar fractures, was compared between the two surgical approaches in three studies [22,23,25]. There were 4 (out of 555) cases of intraoperative fracture in the ABMS group and 5 cases (out of 550) in the DA group. There was no difference in the incidence of intraoperative fractures between the two groups. The heterogeneity was low (OR, 0.79; 95% CI = 0.21~3.02; *p* = 0.73; I^2^ = 0%).

Three studies reported the rate of reoperation. In the ABMS group, 30 patients (out of 706 patients) of reoperation were reported while 35 (out of 1062 patients) were reported in the DA group. There was no difference in the reoperation rate between the two surgical approaches (OR, 1.45; 95% CI = 0.86~2.44; *p* = 0.16). The heterogeneity was low (I^2^ = 0%). 

**Figure 3:**
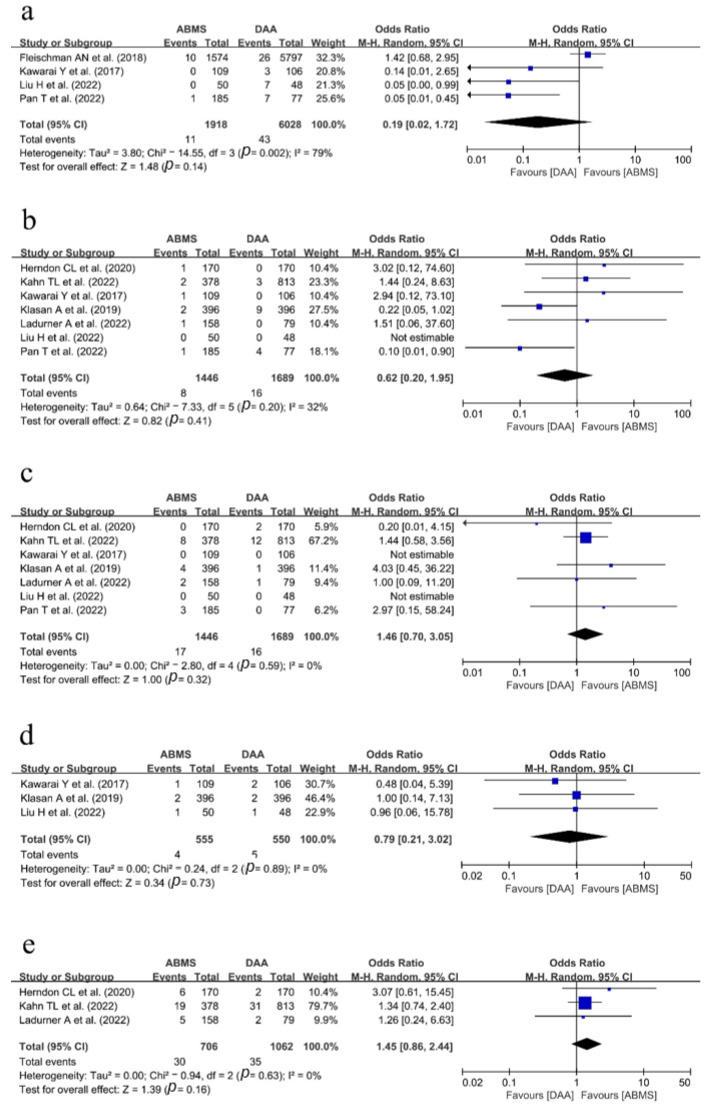
Forest plot showing the postoperative complications between the ABMS approach and the DA approach following hip arthroplasty [18,20,21,22,23,24,25,27]: the number of cases of (**a**) neuropraxia/nerve injury, (**b**) postoperative hip dislocation, (**c**) surgical site infection, (**d**) intraoperative fracture, including trochanter and calcar fracture, and (**e**) reoperation.

A forest plot and further details about postoperative complications are shown in Figure 3.

#### 3.4.3. Implant Position: Cup Inclination, Cup Anteversion, and Stem Alignment

**Figure 4:**
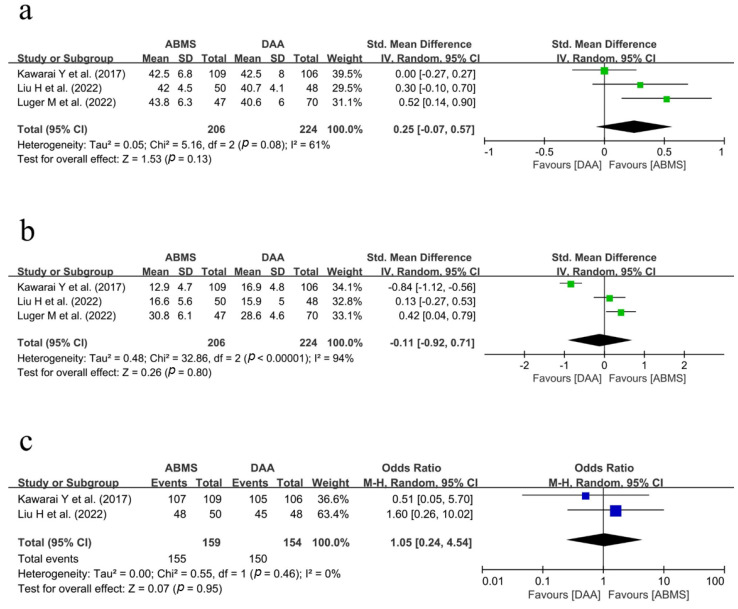
Forest plot showing the postoperative implant position between the ABMS approach and the DA approach following hip arthroplasty [22,25,26]: (**a**) cup inclination, (**b**) cup anteversion, and (**c**) the number of neutral stem alignments.

Three studies compared the postoperative cup position between two surgical approaches. We could extract the data on the rate of neutral stem alignment from two studies. In a pooled analysis, there were no differences between the ABMS group and DA group in terms of cup inclination, cup anteversion, and the rate of stem neutral alignment (cup inclination: SMD, 0.25; 95% CI = −0.07~0.57; *p* = 0.13; I^2^ = 61%; cup anteversion: SMD, −0.11; 95% CI = −0.92~0.71; *p* = 0.80; I^2^ = 94%; stem neutral alignment: OR, 1.05; 95% CI = 0.24~4.54; *p* = 0.95; I^2^ = 0%). A forest plot and further details are shown in Figure 4.

## 4. Discussion

This study demonstrated that the ABMS approach yields comparable results to the DA approach in terms of surgical outcomes, complications, and implant position in all aspects.

In recent years, there has been a substantial increase in research focused on the anterior-based approach, with a clear predominance towards the DA approach [11,28]. A PubMed search for the DA approach showed that less than 20 papers were published annually before 2014, whereas since then, an average of approximately 200 papers per year have been published. 

However, the DA approach has notable disadvantages, including a steep learning curve, concerns about increased complications during the learning period, the risk of lateral femoral cutaneous nerve injury, limitations in implant selection, and difficulties in applying the technique to obese patients [3]. In this regard, the ABMS approach can serve as a viable alternative among the MSAs that can complement these drawbacks. Although the term ABMS has not been firmly established, it is often used synonymously with the anterolateral (AL) approach in hip arthroplasty. However, it is worth mentioning that many researchers mistakenly refer to the direct lateral approach as the AL approach. During the full-text review stage of study selection for this meta-analysis, we encountered five studies [29,30,31,32,33] where the term AL approach was used incorrectly. Consequently, there is a limited number of large-scale prospective studies that genuinely compare the ABMS approach, which can be considered a “new innovative approach [34],” with the DAA. Thus, the objective of this study was to conduct a meta-analysis comparing the ABMS and DA approaches.

Our study findings revealed no statistically significant differences in perioperative surgical outcomes between the ABMS approach and the DA approach. When compared to the traditional PL approach, which is currently the most widely used approach for HA surgery [35], the DA approach has gained consensus in promoting early functional recovery, reducing pain (including the VAS score) and achieving shorter hospital stays, except for the total surgical time [36]. However, it is noteworthy that the ABMS approach is non-inferior to the DA approach in various aspects such as the VAS score, total opioid consumption, length of stay (LOS), and the rate of discharge to the patient’s home. According to a recent study conducted by Kagan et al. [11], the ABMS approach was compared to the minimal incision posterior approach. The study showed comparable results between the ABMS approach and mini-posterior approach in terms of blood loss and surgical time; moreover, it resulted in a shorter LOS by 0.32 days in the ABMS approach compared to the mini-posterior approach. These findings are consistent with the results of the current study, demonstrating that the ABMS approach is as excellent as the traditional DA approach, one of the possible alternative options. Furthermore, this study showed that experienced surgeons could achieve excellent surgical outcomes when transitioning from the mini-posterior approach to the ABMS approach, irrespective of the learning curve. On the other hand, the DA approach often requires a long learning curve, and some studies, like Nairn et al.’s recent research [37], reported that approximately 100 cases of experience are needed for the results to plateau. Taking these results into consideration, the ABMS approach may be a more straightforward alternative for surgeons in clinical practice, compared to the DA approach, as a replacement for traditional approaches.

In terms of postoperative complications, no statistically significant differences were observed between the ABMS approach and the DA approach. A study by Zuskov et al. published in 2022 [38] reported a 10-fold greater risk of lateral femoral cutaneous nerve injury with the DA approach compared to the ABMS approach in their combined retrospective case series and systematic literature review. However, according to our pooled analysis, there was no difference in nerve injury risk between the ABMS and DA approaches. In our opinion, this could be attributed to the differences in the learning curve of each approach. Indeed, according to previous research articles, the incidence of lateral femoral cutaneous nerve injury after the DA approach varies significantly, ranging from 3.37% to 81.0% [39]. A study conducted by Ozaki et al. in 2016 [40] reported that longer surgical times during the DA approach could also impact the occurrence of neurapraxia in the lateral femoral cutaneous nerve. Therefore, we believe that the risk of superficial nerve injury may not significantly differ between the ABMS and DA approaches for experts. But the ABMS approach may offer advantages for beginners in terms of the potential for nerve injury. Additionally, no significant differences were found in terms of dislocation risks, SSI rates, intraoperative fractures, or reoperation rates between the two approaches.

Moreover, no differences were observed between the ABMS and DA approaches in terms of the implant position, including the cup and stem. Fundamentally, both approaches are muscle-sparing. The difference lies in whether the approach is medial or lateral to the tensor fasciae latae. However, below this layer, there is no major difference in the two approaches, leading to the conclusion that after an adequate learning curve, there is no significant difference in the implant position between the two approaches, yielding favorable results. 

Our study had several limitations. First, we were unable to perform various types of subgroup analyses due to the limited number of eligible papers for inclusion. For example, the ABMS approach has the advantage of being applicable in both supine and lateral positions; however, these positions could not be compared separately. Additionally, the DA approach also has different variations, such as the conventional DA approach, performed on a regular table, and the tabled DA approach, performed on a fracture table, but we could not distinguish between them. Second, some of the results of these meta-analyses showed high heterogeneity. This could potentially introduce biases when interpreting the results. Third, a large number of studies included in the research have a retrospective nature. Fourth, the possibility of publication biases should be mentioned. According to the Cochrane guidelines [17], conducting an investigation into publication biases is considered insignificant for meta-analyses that include fewer than 10 studies. This implies that the present study may potentially be susceptible to publication biases. These limitations highlight the need for further research with larger sample sizes and more comprehensive analyses to provide a more robust assessment of the ABMS and DA approaches in hip arthroplasty.

## 5. Conclusions

In current meta-analysis, the ABMS approach demonstrated comparable results to the conventional DA approach in terms of both clinical and radiologic outcomes as well as postoperative complications. Furthermore, the ABMS approach has the advantage of a broader indication and fewer limitations in terms of the surgical position compared to the DA approach. Therefore, the ABMS approach can be even more beneficial as an option within MSAs, surpassing the utility of the DA approach.

## Figures and Tables

**Table 1:** Study design, demographic data, study details, and MINORS scores of included studies.

Author (Year)	StudyDesign	Country	Mean Age	Sex	Type of Hip Surgery	Sample Size		Patient Position	Type of DA	Outcome Investigated	MINORS Score
			(Years)	(%)		ABMS	DA				
Fleischman et al. (2018) [18]	RCS	U.S.	61.9	M (28) F (72)	THA	10	26	ABMS: NA DA: NA	NA	Neuropraxia/nerve injury	17
Gentry et al. (2023) [19]	RCS	U.S.	64.0	M (48) F (52)	THA	207	192	ABMS: NA DA: NA	NA	Op. time, LOS, no. of patients discharged to their homes	17
Herndon et al. (2020) [20]	RCS	U.S.	61.0	M (48) F (52)	THA	170	170	ABMS: Lateral DA: Supine	Conventional	Op. time, VAS score, total opioid consumption, LOS, no. of patients discharged to their homes, dislocation, SSI, ReOp. rate	17
Kahn et al. (2022) [21]	RCS	U.S.	63.5	M (46) F (54)	THA	378	813	ABMS: NA DA: NA	NA	Dislocation, SSI, ReOp. rate	18
Kawarai et al. (2017) [22]	RCS	Japan	67.5	M (9) F (91)	THA	109	106	ABMS: Supine DA: Supine	Conventional	Neuropraxia/nerve injury, Dislocation, SSI, IntraOp. fracture, cup inclination, cup anteversion, stem alignment	18
Klasan et al. (2019) [23]	PSM	Germany	68.7	M (39) F (61)	THA	396	396	ABMS: NA DA: Supine	Tabled	Dislocation, SSI, IntraOp. fracture	17
Ladurner et al. (2022) [24]	PSM	Switz.	85.8	M (29) F (71)	BPHA	158	79	ABMS: Lateral DA: Supine	Tabled	No. of patients discharged to their homes, dislocation, SSI, ReOp. rate	16
Liu et al. (2022) [25]	RCT	China	62.3	M (41) F (59)	THA	50	48	ABMS: Supine DA: Supine	Tabled	Op. time, VAS score, total opioid consumption, no. of patients discharged to their homes, Neuropraxia/nerve injury, dislocation, SSI, IntraOp. fracture, cup inclination, cup anteversion, stem alignment	20
Luger et al. (2022) [26]	RCS	Austria	65.5	M (48) F (52)	THA	47	70	ABMS: Supine DA: Supine	Conventional	Cup inclination, cup anteversion	18
Pan et al. (2022) [27]	RCS	U.S.	63.7	M (42) F (58)	THA	212	79	ABMS: Supine DA: Supine	Tabled	Neuropraxia/nerve injury, dislocation, SSI	17

ABMS, anterior-based muscle-sparing; DA, direct anterior; BPHA, bipolar hemiarthroplasty; LOS, length of hospital stay; NA, not available; No., number; Op., operation; PSM, propensity score matched analysis; RCS, retrospective cohort study; RCT, randomized controlled trial; SSI, surgical site infection; THA, total hip arthroplasty; VAS, visual analogue scale.

## Data Availability

The datasets generated and analyzed during the current study are not publicly available due to the presence of personally identifiable patient information. However, they can be obtained from the corresponding author upon reasonable request.

## Supplementary Materials

The following supporting information can be downloaded at: https://www.mdpi.com/article/10.3390/medicina59081390/s1, Table S1: The literature search algorithm and the results from relevant clinical studies.

## Abbreviations

ABMS, anterior-based muscle-sparing; CI, confidence interval; DA, direct anterior; HA, hip arthroplasty; LOS, length of hospital stay; MSA, muscle-sparing approach; OR, odd ratio; SMD, standard mean difference; SSI, surgical site infection; VAS, visual analogue scale.
