# Supplementary material for: Comparing the Anterior-Based Muscle-Sparing Approach with the Direct Anterior Approach in Hip Arthroplasty: A Systematic Review and Pairwise Meta-Analysis
Source: Medicina (Kaunas). 2023 Jul 29;59(8):1390. doi: 10.3390/medicina59081390 (PMC10456498; doi:10.3390/medicina59081390)
Supplement: Supplementary file 1 [file medicina-59-01390-s001.zip › medicina-2524117-supplementary.pdf]

Table S1. The literature search algorithm and the results from relevant clinical studies.

PubMed (June 7, 2023).

	Search Queries	Articles #
#1	“total hip arthroplasty*”[Title/Abstract]	25,415
#2	“total hip replacement”[Title/Abstract]	9219
#3	Hip Replacement, Total[MeSH Terms]	34,186
#4	#1 OR #2 OR #3	47,036
#5	“watson jones”[Title/Abstract]	256
#6	anterolateral[Title/Abstract]	13,458
#7	able[Title/Abstract]	612,224
#8	“anterior based muscle sparing”[Title/Abstract]	21
#9	#5 OR #6 OR #7 OR #8	625,545
#10	“smith peterson”[Title/Abstract]	101
#11	“direct anterior”[Title/Abstract]	1197
#12	#10 OR #11	1287
#13	#4 AND #9 AND #12	90

Embase (June 7, 2023).

	Search Queries	Articles #
#1	“total hip arthroplasty*”:ti,ab,kw	29,104
#2	“total hip replacement”:ti,ab,kw	12,028
#3	#1 OR #2	38,894
#4	“watson jones”:ti,ab,kw	290
#5	anterolateral:ti,ab,kw	18,165
#6	able:ti,ab,kw	822,481
#7	“anterior based muscle sparing”:ti,ab,kw	19
#8	#4 OR #5 OR #6 OR #7	840,374
#9	“smith peterson”:ti,ab,kw	148
#10	“direct anterior”:ti,ab,kw	1363
#11	#9 OR #10	1499
#12	#3 AND #8 AND #11	92

Cochrane Library (June 7, 2023).

	Search Queries	Articles #
#1	“total hip arthroplasty*”:ti,ab,kw	2
#2	“total hip replacement”:ti,ab,kw	2213
#3	#1 OR #2	2215
#4	“watson jones”:ti,ab,kw	27
#5	anterolateral:ti,ab,kw	788
#6	able:ti,ab,kw	50,772
#7	“anterior based muscle sparing”:ti,ab,kw	0
#8	#4 OR #5 OR #6 OR #7	51,531
#9	“smith peterson”:ti,ab,kw	1
#10	“direct anterior”:ti,ab,kw	201
#11	#9 OR #10	201
#12	#3 AND #8 AND #11	9
